# Phenotypic Screening Reveals Topoisomerase I as a Breast Cancer Stem Cell Therapeutic Target

**DOI:** 10.18632/oncotarget.632

**Published:** 2012-08-31

**Authors:** Fang Zhang, Kristi Rothermund, Sajithlal B. Gangadharan, Yves Pommier, Edward V. Prochownik, John S. Lazo

**Affiliations:** ^1^ Section of Hematology/Oncology, Children's Hospital of Pittsburgh of UPMC, The University of Pittsburgh, Pittsburgh, Pennsylvania; ^2^ Laboratory of Molecular Pharmacology, Center for Cancer Research, National Cancer Institute, NIH; ^3^ Department of Pharmacology and Chemistry, The University of Virginia, Charlottesville, Virginia

**Keywords:** cancer stem cell, Topoisomerase I, small molecule inhibitor, compound library screening

## Abstract

Cancer stem cells (CSCs) are a subpopulation generally thought to be responsible for cancer initiation and progression. Because CSCs are often rare in the total tumor cell population and differentiate rapidly when grown in culture, it has been challenging to uncover compounds that selectively target CSCs. We previously described CSC-emulating cells derived from breast cancer cell lines that maintained a stable undifferentiated state. We optimized a phenotypic assay with these cells and screened 1,280-bioactive compounds, identifying five that preferentially inhibited CSC-like cell proliferation. Using a compound-guided target identification approach, we found high topoisomerase I (Topo I) expression levels in breast CSC-like cells and primary breast CSCs. Structurally unrelated small molecules targeting Topo I preferentially inhibited CSC-like cells. These results illustrate the substantial power of this CSC phenotypic screening platform and promote Topo I as a potential molecular therapeutic target for therapies aimed at expunging CSCs.

## INTRODUCTION

The cancer stem cell (CSC) hypothesis posits that tumors harbor minority populations of undifferentiated “stem cells” capable of making a binary choice between unlimited self-renewal or progression to a differentiated state [1-5]. Analogous to the hematopoietic system [2, 6], CSCs divide infrequently while remaining sufficiently abundant and undifferentiated to ensure regeneration of new CSCs. CSCs can differentiate into so-called “transient amplifying cells” (TACs) with high, but limited, proliferative capacity [1-5]. As a result of this bi-potentiality, CSCs are much more efficient than TACs in surviving serial passage and initiating new tumors. The model predicts that as a result of the disparate proliferative potentials of CSCs and TACs the latter cell type will comprise the bulk of most tumors. Minority populations of CSC-like cells have been identified in many solid tumor types and established cancer cell lines [7-11].

The CSC hypothesis has profound prognostic and therapeutic ramifications. For example, patients with tumors having a higher fraction of breast CSCs would be predicted to have shorter cancer-free intervals, poorer overall survival, and a greater incidence of distant metastasis than individuals with breast tumors containing a low CSC fraction and they do [12, 13]. Because tumors contain mostly TACs, the CSC hypothesis predicts that drug eradication of TACs may produce dramatic tumor regression without providing durable cures if CSCs are not concurrently eliminated. Indeed, taken to its logical extreme, the CSC hypothesis implies that only the CSC population needs to be eliminated because TACs will eventually be lost through attrition [2]. Despite the theoretical attractiveness of the CSC hypothesis, there remains considerable controversy as to the importance of therapeutically targeting CSCs. This is at least partly due to the lack of functional chemical probes and successful clinical agents targeting CSCs.

One strategy to identify small molecules that selectively act on the CSC population would be to target molecules and/or signaling pathways that appear to be critical for CSC survival [14]. Some examples of these self-renewal pathways include: Wnt, Notch, and Hedgehog [14-18], HER2 signaling [19, 20], and macromolecules that control the dialog between CSCs and their microenvironment, such as CXCR1 and IL-8 [19]. An alternative strategy would be to perform unbiased screens with small molecule libraries focused on identifying agents that kill CSCs. The latter tactic requires a large supply of stable and homogenous CSCs to ensure high quality robust screening assays. To date it has not been practical, because CSCs are quite often rare [1, 2, 21, 22]. Moreover, long-term studies are problematic because CSCs tend to differentiate into TACs and thus rapidly lose their CSC-like properties. Finally, current purification techniques only enrich for CSC and do not yield pure populations [2].

As a surrogate, CSC-like cells have been generated by inducing epithelial-to-mesenchymal transition in transformed HMLER breast cancer cells (human mammary epithelial cells overexpressing hTERT, SV40T/t, and H-RasV12) and used in a small molecule compound screen [23, 24]. The transformed HMLER cell model [23] has some limitations, however. First, the HMLER cell line was derived by enforcing the expression of SV40 T-antigen and mutant Ras in primary mammary epithelial cells. Very few human breast cancers are associated with Ras oncogene mutations and none express T-antigen. Second, the starting cells are already fully differentiated at the time of tumorigenic transformation and their final phenotype, while undifferentiated, was more consistent with cells undergoing an epithelial-mesenchymal transition than with stem cells. Finally, the HMLER cells have enforced down-regulation of E-cadherin, which may or may not have led to the emergence of true CSCs that faithfully recapitulate those arising from clinical specimens.

As an alternative, we derived cell populations from three different human cell lines that emulate many of the properties of breast CSCs and can be maintained in an undifferentiated state for extended periods [25]. These “blocked” CSC-emulating cells can be distinguished from non-CSCs by their resistance to chemotherapeutic drugs, hypoxic and acidotic conditions, by their transcriptional profiles, and by their superior tumor-initiating activities [25]. Moreover, we observed that the CSC-initiated tumors were composed almost exclusively of pure CSCs, thus providing incontrovertible evidence that they retain their undifferentiated state *in vivo* [25]. These blocked CSCs are the only cells known to us that are derived directly from naturally arising human tumors and, thus, represent unique reagents for studying CSC-like properties in a homogeneous, non-differentiating state and may provide a functional tool for use with high throughput screening approaches to identify novel CSC-selective chemical probes and therapeutic leads.

In the current study, we describe the development and optimization of an assay suitable for high throughput screening. We then used the assay to interrogate the widely available Library of Pharmaceutical Active Compounds (LOPAC), which includes 1,280 known bioactive small molecules to identify compounds that selectively target breast CSCs. We discovered five small molecules that preferentially inhibit the growth of CSC-like cells, one of which was β-lapachone. This compound has a number of reported activities including the generation of reactive oxygen species and inhibition of topoisomerase I (Topo I). Topo I is an attractive actionable molecular cancer target because there are experimental and clinically used Topo I inhibitors. Remarkably, we observed high Topo I expression in breast CSC-like cells and primary breast CSCs and found other Topo I inhibitors from distinct chemical classes also exhibited preference for the CSC-like cells. Our data suggested that Topo I might be a potential CSC marker and thus an attractive therapeutic target.

## RESULTS

### Development and optimization of the high throughput CSC assay

The establishment of the CSC-like and non-CSC-like cells were previously described [25]. For simplicity we have adopted the following nomenclature: BC1A and BC1B for the MCF-7-derived CSC-like and non-CSC-like populations, respectively; BC2A and BC2B for the MDA-MB-231-derived CSC-like and non-CSC-like populations, respectively; and BC3A and BC3B for the MDA-MB-453-derived CSC-like and non-CSC-derived populations. We initially focused on examining the two CSC pairs, BC1 and BC2 cells, because they are thought to be represented of stage IV adenocarcinomas with BC1 being estrogen and progesterone receptor positive and Her2/neu^low^ and BC2 being negative for estrogen and progesterone receptor and as well as Her2/neu (*i.e*., triple negative). Moreover, they are maintained in the same culture conditions. Using a simple, economical, and previously published [25, 26] 384-well metabolic alamar blue assay, we observed a time- and cell seeding-dependent increase in endpoint signal for cell viability. For both the BC1 and BC2 cell pairs we found 72 hr to be the optimal incubation time with the greatest signal to background level. The optimal 384-well cell plating density for BC1A and BC1B cells was 1,000 cells/well and similar results were obtained with the BC2 cell pair (Supplemental Fig. 1). Both sentinel CSC pairs tolerated DMSO concentrations of ≤1%. Plate variation was examined with BC1 and BC2 pairs using different volumes of medium in each well (30, 50 and 75 μl) for the 72 hr incubation period to minimize edge effects. Surprisingly, the well volume producing the best coefficient-of-variation (<5%) was obtained with 30 μl. This volume had an edge/center ratio of 0.93. We also conducted three-day variability tests to determine the signal window and the Z’-factors with the minimum (MIN) (a-MEM with 0.05% DMSO) and maximum (MAX) (5 μM doxorubicin, 0.05% DMSO) controls. We found <10% variability with the three day experiments, signal-to-background of >8 and Z’-factors >0.5. One of these three-day variability results with BC1A cells with a Z’-factor of 0.83 is shown in Supplemental Figure 2.

### LOPAC library screen identifies β-lapachone as a candidate inhibitor of breast CSC-like cell growth

**Figure 1:**
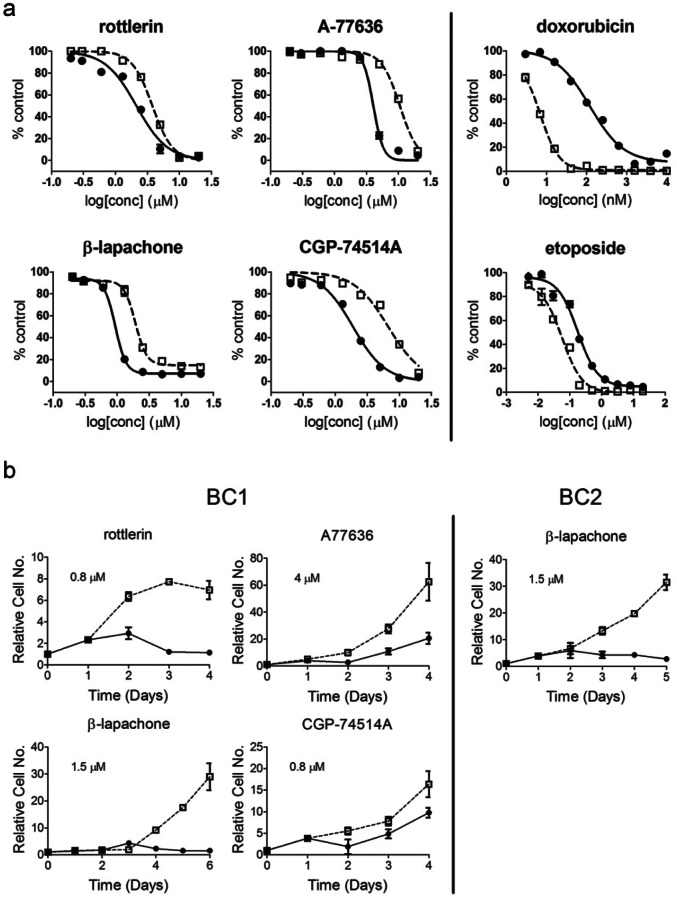
Concentration-dependent curves for the four compounds identified as selective against BC1A cells (a) BC1A and BC1B cells were treated with rottlerin, A77636, β-lapachone, CGP74514A, doxorubicin, or etoposide at the indicated concentrations. CSC-like cells (●); non-CSC-like cells (□). Cell viability was accessed using alamar blue after 72 hr. The fluorescence readout (RFU) was normalized to in-plate control to calculate the percent control. Each concentration was tested in quadruplicate and the data are the mean ± SD; each drug panel is representative of three experiments with similar results. (b) Growth curves for the four CSC selective compounds. BC1A and BC1B cells were treated with rottlerin (0.8 μM), A77636 (4 μM), β-lapachone (1.5 μM), or CGP74514A (0.8 μM) for 4 or 6 days. BC2A and BC2B cells were treated with β-lapachone (1.5 μM) for 5 days. We used drug concentrations that yielded the maximum selective difference in growth inhibition from Figure 1a. Viable cells were counted using the Vi-Cell Cell Viability Analyzer and the number of viable cells on day 0 was used to normalize the relative cell number for the subsequent days. CSC-like cells (●); non-CSC-like cells (□). Experiments were performed in triplicate and the data were presented as the average relative cell number ± SD. Each growth curve was repeated three times with similar results and one representative curve is shown.

We next examined the performance of the CSC-like and non-CSC-like pairs in a high throughput screening platform using a 1,280 small molecule LOPAC set. Both pairs of cells exhibited reproducible results when tested on separate days with Z’-factors between 0.52 and 0.89 (Supplemental Fig. 3). From this library we identified 35 compounds that caused >40% growth inhibition of BC1A cells (Table 1). At the concentration tested (5 μM), most of the compounds produced similar inhibition of both cell populations. Five compounds, however, caused preferential growth inhibition of BC1A cells compared to BC1B cells. These were: A77636 (9.7-fold, p<0.02), rottlerin (3.0-fold, p<0.001), β-lapachone (1.4-fold, p<0.004), CGP-74514A (1.3-fold, p<0.02), and C-14 linker dequalinium analog (1.3-fold, p<0.01) (Table 1). There also were several small molecules, namely disopyramide phosphate, the methotrexate enantiomer ametopterin, and cytosine-1-b-D-arabinofuranoside, to which CSC-like cells were more resistant. We also used the identical screening approach with the BC2 cell pair and identified six compounds in the LOPAC collection that caused a preferential inhibition of BC2A cells compared to the BC2B cells (Table 2). Complete concentration-response studies indicated A77636, rottelerin, β-lapachone, and CGP74514A were approximately 3-4 fold selective for BC1 CSC-like cells compared to the non-CSC-like counterparts at multiple concentrations (Fig. 1a). In comparison, the CSC-like cells were more resistant to doxorubicin and etoposide, indicating that the preferential inhibition of CSC-like population by the identified compounds was not due to artifact induced by the cell model or experiment design. Pharmacological kinetic studies also suggested rottlerin, A77636 and β-lapachone rapidly suppressed any increase in the number of CSC-like cells (Fig. 1b).

**Table 1:** The 35 compounds at 5 μM that caused >40% growth inhibition of BC1A cells These are the mean results from three independent screens. The asterisks indicate compounds with preferential inhibition for BC1A cells compared to BC1B cells (Student t test, p < 0.05).

Compound	BC1A cells		BC1B cells	
	% Inhibition	SD	% Inhibition	SD
Idarubicin	100.4	0.1	100.3	0.1
Emetine dihydrochloride hydrate	100.4	0.3	98.6	1.8
Mitoxantrone	99.3	0.5	99.2	0.1
Ammonium pyrrolidinedithiocarbamate	95.0	1.7	101.8	0.3
Brefeldin A	87.0	0.9	85.3	0.40
Quinacrine dihydrochloride	84.9	5.0	74.3	5.5
Ouabain	83.7	2.1	77.7	1.4
Thapsigargin	81.6	2.2	76.3	3.6
β-Lapachone *	81.3	3.0	57.4	4.6
Tetraethylthiuram disulfide	81.2	9.8	99.2	0.9
(S)-(+)-Camptothecin	80.3	1.9	73.6	0.8
C-14 linker dequalinium analog *	79.9	1.6	59.9	7.7
Pacletaxel	68.9	0.6	62.5	2.8
CGP-74514A *	78.0	1.0	57.7	9.5
Vincristine sulfate	77.7	0.5	68.3	3.3
Dihydroouabain	77.4	1.5	71.2	1.2
Vinblastine	77.2	0.3	62.8	6.5
Podophyllotoxin	76.2	1.0	62.7	1.8
Colchicine	75.3	0.1	60.7	2.9
Methotrexate	72.9	2.5	74.7	1.6
Niclosamide	65.1	17.3	65.7	12.9
Calcimycin	64.5	5.1	73.4	1.5
Diphenyleneiodonium chloride	63.7	2.8	60.5	12.0
Cytosine-1-β-D-arabinofuranoside	63.1	5.8	82.6	0.2
Rotenone	61.3	5.0	58.8	4.3
A-77636 *	59.9	6.3	6.2	1.3
Aminopterin	59.5	4.6	72.3	1.6
7-Chloro-4-hydroxy-2-phenyl-1,8-naphthyridine	58.6	2.7	52.7	2.1
Amsacrine	56.1	2.1	68.0	2.5
Ancitabine	52.6	10.5	57.9	14.7
Etoposide	51.2	0.6	67.0	1.1
Rottlerin *	46.2	2.9	15.5	1.7
(-)Amethopterin	45.6	0.8	60.2	5.1
Disopyramide phosphate	44.0	1.2	68.9	2.2
Nocodazole	42.5	18.0	53.6	3.2

**Table 2:** The 22 compounds that at 5 μM caused >40% growth inhibition of BC2A cells These are the mean results from three independent screens. The asterisks indicate compounds with preferential inhibition for BC2A cells compared to BC2B cells (Student t test, p < 0.05).

Compound	BC2A cells		BC2B cells	
	% inhibition	SD	% inhibition	SD
Idarubicin	101.5	0.5	103.5	0.66
Mitoxantrone	99.6	0.9	100.2	0.88
β-Lapachone*	81.4	1.6	51.7	1.29
C-14 linker dequalinium analog	74.0	2.7	78.6	3.58
Ammonium pyrrolidinedithiocarbamate*	73.1	13.9	31.2	11.1
Emetine dihydrochloride hydrate	71.1	2.6	60.2	1.8
Quinacrine	70.8	8.8	50.5	8.8
Diphenyleneiodonium chloride	70.6	2.8	70.5	1.6
Ouabain	62.7	3.2	58.7	5.3
2,3-Dimethoxy-1,4-naphthoquinone*	61.0	8.4	42.5	5.4
Brefeldin A	54.6	3.3	57.4	4.4
Cytosine-1-β-D-arabinofuranoside *	53.9	9.1	23.9	4.7
Calcimycin	50.2	5.6	40.4	6.0
Colchicine	47.2	5.2	34.2	3.3
Tetraethylthiuram disulfide*	46.5	8.8	1.1	4.9
(S)-(+)-Camptothecin*	46.5	5.1	24.1	4.5
Dequalinium dichloride	45.1	6.2	72.4	3.1
Thapsigargin	45.0	1.4	47.9	2.6
Doxycycline	43.5	0.6	46.6	5.6
Apomorphine	42.1	12.6	29.5	8.4
Demeclocycline	42.0	5.9	29.9	8.8
NSC 95397*	40.9	7.2	24.4	6.9

### Chemotype independent Topo I inhibitors preferentially inhibit CSC-like cells

**Figure 2:**
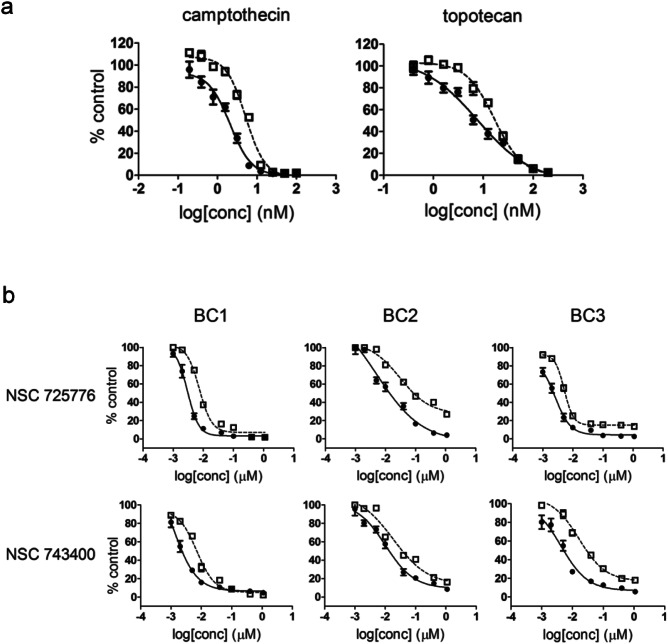
CSC selective growth inhibition by Topo I inhibitors (a) BC1A and BC1B cells were treated with camptothecin and topotecan at indicated concentrations. Cell viability was accessed using the alamar blue after 72 hr. The fluorescence readout was normalized to in-plate control to calculate the percent of control. Each concentration was tested in quadruplicate and the data were presented as mean ± SD. Each curve was repeated three times with similar results and one representative curve is shown. CSC-like cells (●); non-CSC-like cells (□). (b) BC1A, BC1B, BC2A, BC2B, BC3A, and BC3B cells were treated with NSC 725776 or NSC 743400 at indicated concentrations and as described in panel A. CSC-like cells (●); non-CSC-like cells (□).

Because β-lapachone displayed selectivity for both BC1A and BC2A populations, has previously reported preclinical antitumor activity, and has been the subject of clinical trials [27-30], we focused on one of its putative molecular targets to guide our efforts to understand the ideal selective CSC drugs, while recognizing that the true molecular target of β-lapachone remains somewhat controversial. The first identified molecular target for β-lapachone, however, was Topo I [31], an enzyme critical in DNA replication and transcription [32] that is often elevated in the tumor cells compared to normal cells [33-35]. Thus, we first tested several other previously identified Topo I inhibitors and found that camptothecin and topotecan were 2-3 fold more potent against BC1A cells compared to BC1B cells (Fig. 2a). The preferential inhibition of CSC-like cells was surprising because both camptothecin and topotecan are substrates for the drug efflux pump, ATP-binding cassette sub-family G member 2 (ABCG2), and CSCs are reported to have higher level expression of ABCG2 [36-40]. Indeed, we observed increased levels of ABCG2 in some CSC-like cells [25]. Therefore, we tested two other Topo I inhibitors that are structurally unrelated to β-lapachone and are not substrates for ABCG2. Both NSC 725776 and NSC 743400 were more toxic to BC1A and BC2A cells compared to BC1B and BC2B cells, respectively (Fig. 2b).

### Breast CSC-like cells have a higher expression of Topo I

**Figure 3:**
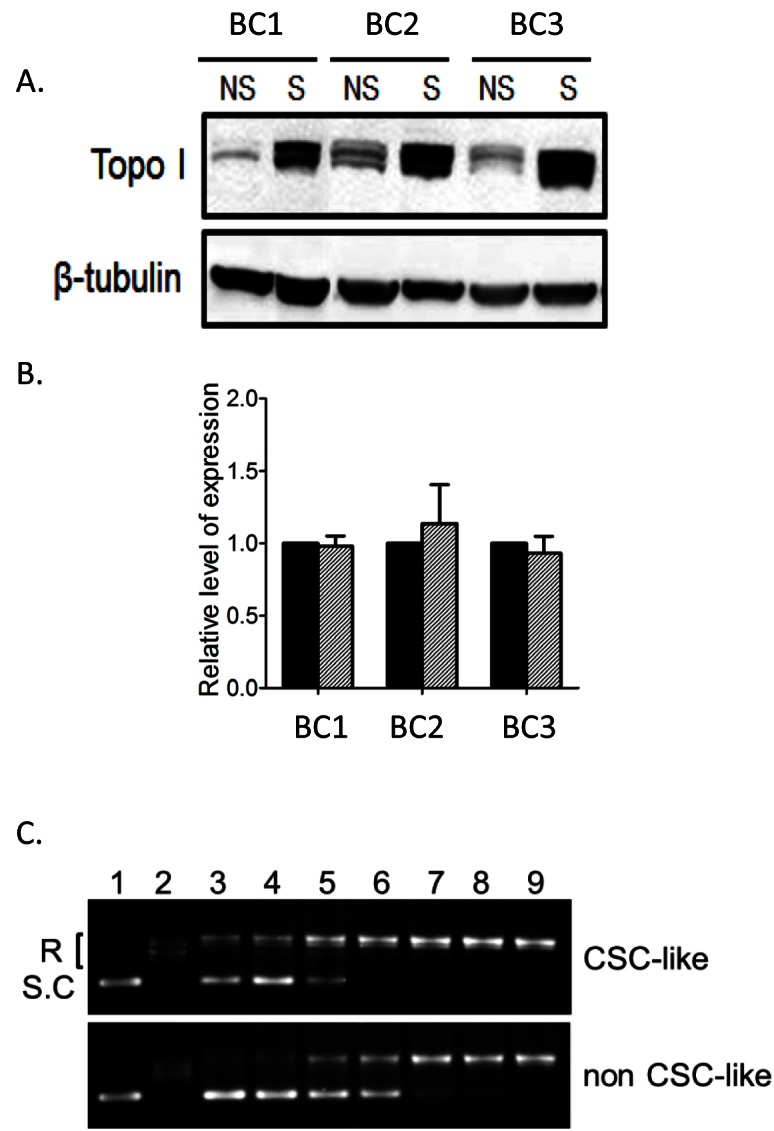
Elevated Topo I levels and activity in breast CSC-like cells (A) Western blots for Topo I protein in CSC-like (S) and non-CSC-like (NS) cells from BC1, BC2 and BC3 cells. β-tubulin was used as a loading control. (B) Topo I mRNA levels were determined in the three CSC pairs by quantitative real-time RT-PCR. Human GAPDH was used as an internal control. Data were normalized to GAPDH mRNA level first. Topo I mRNA level in CSC-like cells were then normalized to non-CSC-like cell Topo I level to calculate the fold change. Each PCR reaction was performed in triplicate, and the data were presented as the average fold change ± SD. CSC-like cells = hatched bars; non-CSC-like cells = black bars. (C) Cell extracts from BC1A and BC1B cells were serial diluted 2-fold before the activity assay. Extracts were incubated with supercoiled plasmid substrate DNA at 37°C for 30 min. After incubation, DNA samples were separated by electrophoresis on 1% agarose gel and stained with ethidium bromide. The supercoiled DNA (S.C, lane 1) and relaxed DNA (R, lane 2) samples are shown for reference. Lane 3 to 8 were DNA incubated with diluted extract, corresponding to 1:64, 1:32, 1:16, 1:8, 1:4, and 1:2 dilutions. Lane 9 was DNA incubated with undiluted extract.

We next examined if Topo I expression levels were different in the CSC-like cells and non-CSC-like cells. Topo I protein levels in protein lysates obtained from BC1A, BC2A and BC3A breast cancer cells were increased compared to their non-CSC-like counterpart (Fig. 3A). Interestingly, the mRNA levels in CSC-like and non-CSC-like cells were comparable (Fig. 3B), which may explain why this molecular target was previous unnoticed in CSCs. We examined cells for their Topo I enzyme activity by incubating cell extracts with supercoiled plasmid DNA substrate at different titrations to determine the minimum amount of cell extract required for completely converting the supercoiled DNA to relaxed DNA. As illustrated in Figure 3C, CSC-like cell extracts completely relaxed the DNA substrate. In contrast, supercoiled DNA was still detectable on the gel when incubated with a 4-fold higher concentration of non-CSC-like cell extract (Fig. 3C). Thus, Topo I activity appeared to be elevated in the CSC-like cells by at least 4-fold in the absence of elevated mRNA levels.

### Primary breast CSCs also express higher levels of Topo I

**Figure 4:**
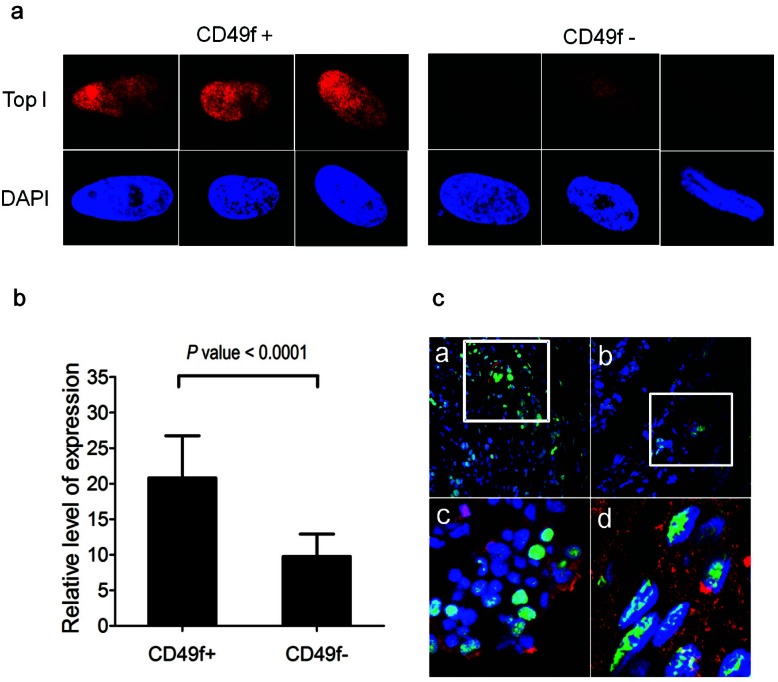
Higher Topo I expression in primary breast CSCs (A) CSCs and non-CSCs were isolated from primary tumors using CD49f as a marker and stained with anti-Topo I antibody (red) and DAPI (blue). Cells were directly examined by fluorescence microscopy and Topo I was found expressed exclusively in the nuclei. CSC (CD49f^+^ cells) had much higher level of Topo I staining compared to non-CSC (CD49f^−^ cells). Images showed 3 representative cells of CSC and non-CSC. (B) Topo I mean fluorescence intensity was higher in CSC-like cells. Fluorescence intensity of each cell was quantified using the Image J software. 41 CD49^−^ cells and 50 CD49f^+^ cells were measured and mean fluorescence intensities were calculated. (C) Co-localization of breast CSC marker ALDH1 and Topo I in frozen tumor sections. Frozen sections of 19 breast tumors were stained with DAPI (blue), anti-Topo I antibody (green), and anti-ALDH1 antibody (red). Only a small subset of tumor cells expressed ALDH1 and these cells formed clusters (a and b). Co-localization of ALDH1 and Topo I was evident in 15 out of the 19 tumors we examined. Panel c and d are enhanced magnifications.

We next isolated CSCs from primary tumors to test whether Topo I level were also higher in primary breast CSCs. Primary breast tumors were enzymatically digested to generate single cell suspensions and sorted by flow cytometry using CD49f as a CSC marker. CSCs (CD49f^+^) and non-CSCs (CD49f^−^) cells were cultured on coverslips for 2 days prior to fixation and staining with Topo I antibody and DAPI. As shown in Figure 4A, Topo I was found exclusively in the nuclei of both cell types, although CSCs had 2-fold higher levels of Topo I staining compared to non-CSCs (Fig. 4B). We also investigated Topo I expression in breast CSCs from frozen tumor sections. To identify CSCs, we stained the frozen sections with antibody against a CSC marker, aldehyde dehydrogenase 1 (ALDH1) [41], and found that ALDH1-positive cells existed in the tumors as clusters with considerable inter-tumor variability in the percentage of ALDH1-positive cells. ALDH1-positive cells were detected in 14 of the 19 tumor sections we examined. All 14 tumors with detectable ALDH1-positive cells showed evidence of co-localization of ALDH1 and Topo I (Fig. 4C). There was also, however, a population of ALDH1-expressing cells that did not have high expression of Topo I. Consequently, our data suggested Topo I expression is not necessarily co-regulated with ALDH1 expression in primary tumor CSCs.

## DISCUSSION

In this initial high throughput screen, we focused on the LOPAC collection because it contains an attractive selection of approved clinical drugs and other small molecules with known cellular targets. It is encouraging the even from this limited screen different compounds were identified with the two CSC-like and non-CSC-like pairs, indicating that CSCs from different cell lines may have distinct key cellular targets/pathways even though they share many common CSC properties. Our experience with one compound, β-lapachone, illustrates that potential for identifying new molecular targets for breast CSCs. β-Lapachone was ~4-fold more selective for the BC1A and BC2A cells relative to their non-CSC-like counterparts. β-Lapachone is an extensively studied naphthoquinone nature product with documented antineoplastic effects in preclinical models [42]. It has not achieved clinical success, however, which may reflect its modest potency and its limited aqueous solubility that complicates formulation and delivery. A proposed mechanism for β-lapachone mediated cell death is via activation of a futile cycling by the cytoplasmic two-electron reductase NAD(P)H:quinone oxidoreductase, also known as NQO1[43]. NQO1 reduces β-lapachone to an unstable hydroquinone that rapidly undergoes a two-step oxidation reverting to the parent compound and perpetuating a futile redox cycle with the generation of reactive oxygen species that damage DNA, activate poly(ADP-ribose) polymerase and deplete cellular NAD^+^ and ATP. β-Lapachone is toxic, however, even to cells that lack highly functional NQO1, such as MDA-MB-231 cells, which harbor a polymorphism that markedly reduces enzyme activity. Thus, we were obligated to consider other molecular targets. β-Lapachone has been studied extensively and, as might be expected with a naphthoquinone, it inhibits numerous enzymes at least *in vitro*. While we focused on Topo I because it was the first proposed target of β-lapachone [31] and there are clinically used drugs available, the PubChem database (http://pubchem.ncbi.nlm.nih.gov/) lists other potential targets including Bloom's syndrome helicase, RecQ-like DNA helicase, thioredoxin reductase, histone lysine methyltransferase, vitamin D receptor and cytochomes P450 2C9, 3A4, 2D6, and 2C19. Some of these macromolecules may also be CSC-specific targets worthy of further investigation.

The CSC-like cells were 2-3 fold more sensitive to camptothecin and topotecan. The two indenoisoquinolines, NSC 725776 and NSC 743400, which are currently in clinical trials, also displayed selective growth inhibition to the CSC-like population. Compare to camptothecin and its derivatives, NSC 725776 and NSC 743400 are chemically stable, form persistent Topo I-DNA complexes and have potent antitumor activity in preclinical models [44, 45]. The ability to target the CSC population adds to the attractiveness of these compounds for further development.

Our study highlights how an unbiased phenotypic screen can lead to the recognition of a previously unsuspected CSC molecular target. The omission of Topo I as a CSC molecular target may reflect a previous focus on gene expression profiling; we saw no increase in Topo I mRNA. Interestingly, studies in lower organisms, namely *Arabidopsis thaliana*, however, link Topo I to stem cell stability and cellular memory [46]. Topo I is an enzyme that resolves topological DNA stress during replication and transcription [32, 45, 47]. Previous studies suggest that both proliferating and quiescent tumor cells have up-regulated, higher levels of Topo I compared to normal cells [48]. We found Topo I expression and activity were higher in the CSC-like cells compared to the non-CSC-like counterpart and elevated levels of Topo I were seen in CSC from primary tumors. It is notable that Topo I inhibitors have to date not been found useful in the treatment of human breast cancer, which may reflect the involvement of other resistance factors for the existing clinically used drugs. Thus, development of structural distinct Topo I inhibitors, like NSC 725766 and NSC 743400 appears warranted. While we recognize phenotypically distinct populations of CSC may exist in patient-derived tumors from different organ sites, we propose Topo I might not only be a valuable molecular target for some CSC but also a useful adjunct for CSC diagnostic reagents.

## METHODS

### Cell culture

CSC-like and non-CSC-like cells were previously described [25] as being derived from MCF-7, MDA-MB-231, and MDA-MB-453 cells. Briefly, parental cells were stably transfected with a plasmid encoding GFP driven by a 4.0 kb segment of the Oct 3/4 promoter and selected using G418. GFP-positive (CSC-like) and GFP-negative (non-CSC-like) cells were separated using flow cytometry. These well-characterized cell pairs [25] will be referred throughout this report as BC1A and BC1B for the MCF-7-derived CSC-like and non-CSC-like populations, respectively, BC2A and BC2B for the MDA-MB-231-derived CSC-like and non-CSC-like populations, respectively, and BC3A and BC3B for the MDA-MB-453-derived CSC-like and non-CSC-derived populations. BC1A, BC1B, BC2A and BC2B cells were maintained in α-modified Eagle's Minimal Essential Medium (α-MEM) supplemented with 10% fetal bovine serum (FBS), 1 mM sodium pyruvate, 100 μM nonessential amino acids, 100 units/ml penicillin G, and 100 μg/ml streptomycin. BC3A and BC3B cells were maintained in Dulbecco's Modified MEM (D-MEM) supplemented with 10% FBS, 1 mM sodium pyruvate, 100 μM nonessential amino acids, 100 units/ml penicillin G, and 100 μg/ml streptomycin. All medium and chemical supplements were obtained from Mediatech (Manassas, VA) and serum supplement was obtained from Atlanta Biological (Atlanta, GA). The cellular origins of BC2A, BC2B, BC3A and BC3B have been confirmed by genotyping but BC1A and BC1B have not.

### Compound conformation assays

For the confirmation assays, compounds were purchased from Sigma Aldrich unless otherwise noted. NSC 725766 and NSC 743400 [39, 49] were obtained from the National Cancer Institute. For the concentration-response curves, compounds were diluted in α-MEM and a two-fold serial dilution was performed in quadruplicate. For the 10-point concentration-response study, each curve was independently repeated at least 3 times. For some compounds, additional growth curve experiments were performed in 96-well plates in which 1000 cells were plated per well (200 μl) and allowed to attach for 24 hr prior to the addition of compounds. Cells were treated with compounds for 4-6 days. Cells were then detached with trypsin/EDTA and resuspended in 1 ml Phosphate Buffered Saline (PBS) and counted by Vi-Cell Cell Viability Analyzer (Beckman Coulter, Inc. Miami, FL).

### Western blotting and immunochemistry staining

Cells in 6-well plates were washed 3 times with ice-cold PBS and scraped into a modified radioimmunoprecipitation buffer [50]. Lysates were incubated on ice for 30 min and vortexed every 5 min, and then cleared by centrifugation at 13,000 xg for 20 min. Protein concentrations were determined using the Bio-Rad Protein Assay (Bio-Rad Laboratories, Hercules, CA). Protein lysates (30 μg) from each sample were loaded and resolved on 8% SDS-polyacrylamide gels and transferred to nitrocellulose membranes. Membranes were probed with Topo I antibody (Abcam, Cambridge, MA). β-Tubulin was used as a loading control.

Primary human breast cancer CD49f^+^ and CD49f^−^ cells were obtained from Dr. Jean Latimer at the University of Pittsburgh. Cells were cultured on glass coverslips for at least 2 days and fixed with 4% para-formaldehyde. Cells were then permeabilized with 0.1% Triton X-100 and blocked with 1% BSA. Cells were incubated with rabbit polyclonal anti-human Topo I antibody (Sigma-Aldrich) for 2 hr at room temperature and with Alexa fluor 594 labeled anti-rabbit secondary antibody (Invitrogen) for 1 hr. Nuclei were counter-stained with 0.05% DAPI. Images were captured with an Olympus FluoView FV1000 confocal microscope (Olympus, Center Valley, PA). Fluorescence intensity was quantified using the ImageJ software (NIH). In total, 41 CD49^−^ cells and 50 CD49f^+^ cells were measured to calculate the mean fluorescence intensity.

Human breast tumor samples were obtained from the Tissue Bank of the University of Pittsburgh. Frozen sections were prepared by the Children's Hospital of Pittsburgh histopathology facility. Tissue slides were fixed with 4% para-formaldehyde and permeabilized with 0.5%Triton X-100. Slides were then blocked with 1% BSA for 1 hr and incubated with rabbit polyclonal anti-human Topo I (Sigma) and mouse monoclonal anti-human ALDH (BD Biosciences, Franklin Lakes, NJ) primary antibodies for 2 hr at room temperature. The slides were then washed with PBS and treated with Alexa fluor 488-labeled anti-rabbit and Alexa fluor 594-labeled anti-mouse secondary antibodies for 1 hr; cell nuclei were counter-stained with DAPI. Slides were photographed using an Olympus Fluoview 1000 confocal microscope.

### Topo I activity assay

Topo I extracts from CSC-like or non-CSC-like cells were prepared following a protocol provided by TopoGen, Inc. (Port Orange, FL). Cells were scraped into medium and centrifuged at 800 xg for 3 min at 4^°^C. Cell pellets were re-suspended in 4 ml TEMP buffer (10 mM Tris-HCl, 1 mM EDTA, 4 mM MgCl_2_, 0.5 mM phenylmethylsulfonyl fluoride, pH 7.5), centrifuged again, re-suspended in 3 ml TEMP buffer, and placed on ice for 10 min. Cells were then homogenized in a glass tissue dounce tube for 8 strokes. Cell nuclei was centrifuged at 1500 xg for 10 min at 4^°^C, re-suspended in 1 ml cold TEMP, transferred to an Eppendorf microfuge tube, and centrifuged at 15,000 xg (4^°^C) for 2 min. The nuclear pellet was re-suspended in 50 μl TEP buffer (TEMP but lacking MgCl_2_) and 50 μl 1M NaCl, placed on ice for 60 min and centrifuged in a microfuge at 15,000 xg 4^°^C for 15 min. Protein concentrations of extracts from CSC-like and non-CSC-like cells were determined using the Bio-Rad Protein Assay and adjusted to equal protein concentrations. Topo I activity was assayed using Topo I Assay Kit (TopoGen) following the manufacturer's instructions. To accurately estimate the Topo I activity, we prepared extracts at two-fold dilutions, namely, 1:2, 1:4, 1:8, 1:16, 1:32, and 1:64. Briefly, 12 μl distilled water, 2 μl 10x assay buffer, 1 μl supercoiled DNA and 4 μl Topo I extract (original extract or diluted) were added to a micro-centrifuge tube and incubated for 60 min at 37^°^C. After adding 5 μl stop loading dye, the sample was loaded to a 1% agarose gel, subjected to electrophoresis at 2 volts/cm and the gel stained with 0.5 μg/ml ethidium bromide for 20 min at room temperature. Gels were destained with distilled water for 20 min and photographed using FujiFilm LAS-3000 (FujiFilm, Tokyo, Japan).

## Supplementary Figures


