# Supplementary material for: Phenotypic Screening Reveals Topoisomerase I as a Breast Cancer Stem Cell Therapeutic Target
Source: Oncotarget. 2012 Aug 31;3(9):998–1010. doi: 10.18632/oncotarget.632 (PMC3660065; doi:10.18632/oncotarget.632)
Supplement: Supplementary file 1 [file oncotarget-03-998-s001.pdf]

Phenotypic Screening Reveals Topoisomerase I as a Breast Cancer Stem Cell Therapeutic Target – Zhang et al

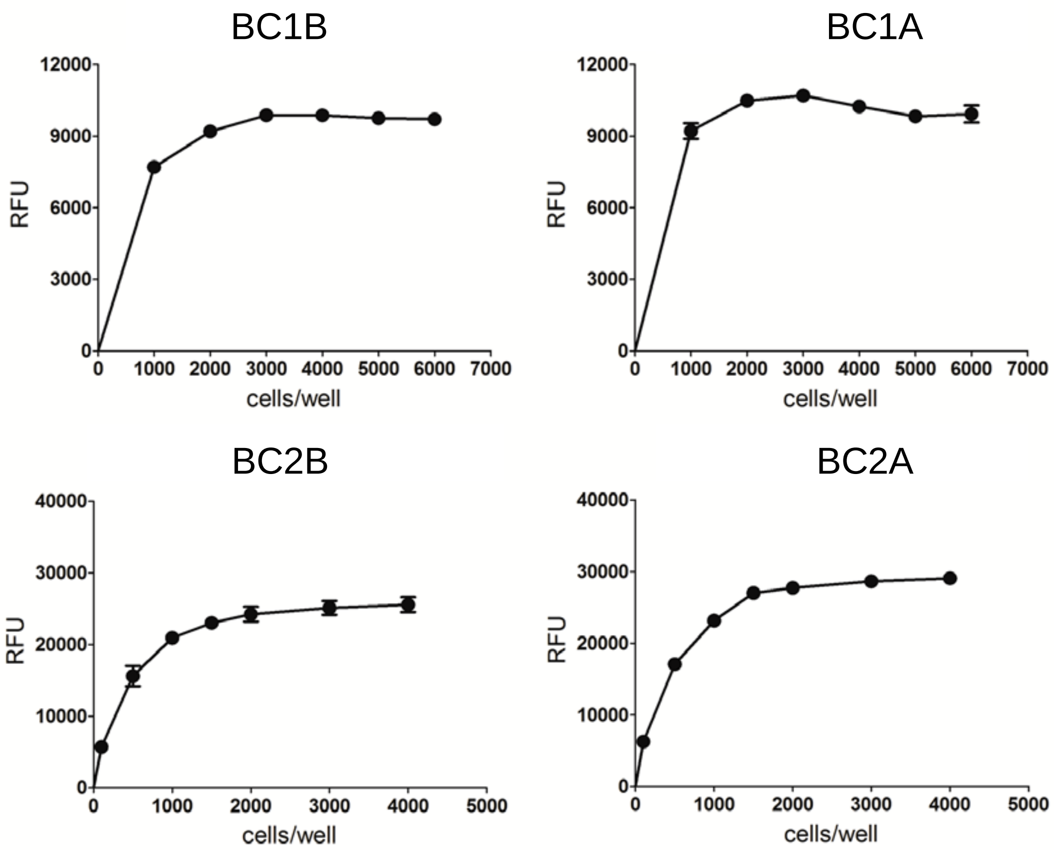

Supplemental Figure 1: Optimization of cell seeding of CSC-like and non-CSC-like cell populations. Cells were seeded in 384-well plates and incubated as previously described for 72 h and cell viability determined with a fluorometric resazurin based assay [25].

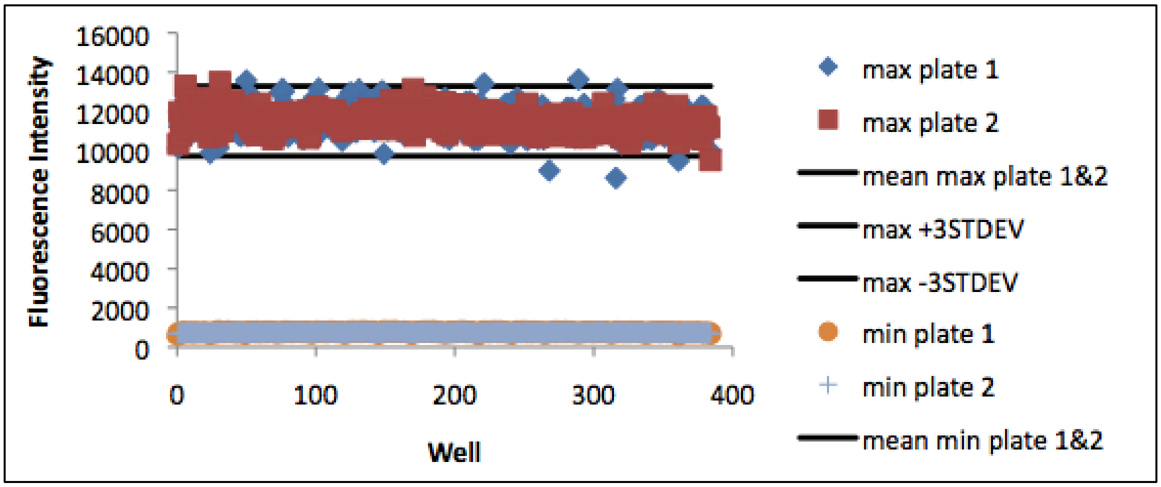

Supplemental Figure 2: Assay signal window Z'-factor determination for CSC-like cell populations. This is representative of one of three results from a three-day variability assay. MCF-7 CSC-like cells were seeded in 384-well plates and incubated as previously described for 72 h and cell viability determined with a fluorometric resazurin based assay [25]. The minimum (MIN) inhibition was determined with α-MEM containing 0.05% DMSO and the maximum (MAX) inhibition was 5 μM doxorubicin in 0.05% DMSO.

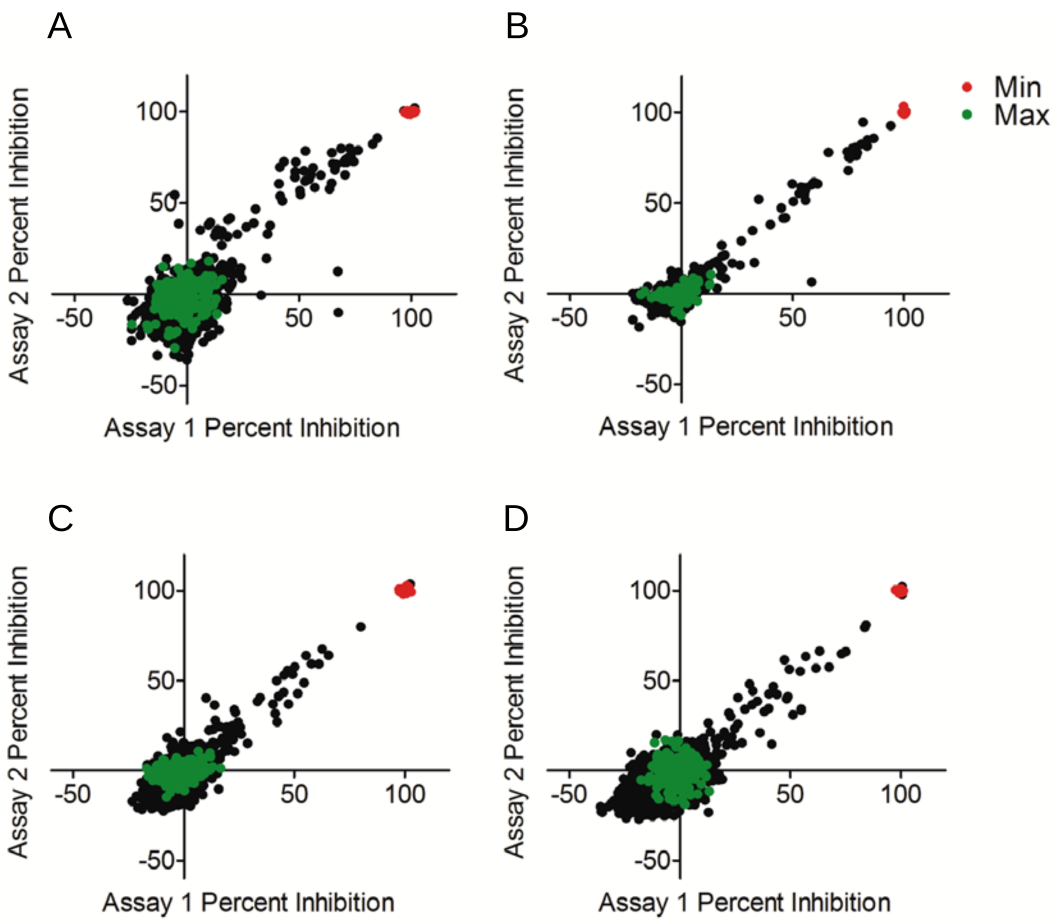

Supplemental Figure 3: Performance of alamar blue assay with CSC pairs using LOPAC validation set. Studies were performed with the 1,280 LOPAC set on 2 separate days. The slope values were: MCF-7 non-CSC-like 0.95; MCF-7 CSC-like 0.75; MDA-MB-231 non-CSC-like 0.75; MDA-MB-231 CSC-like 0.89, respectively.
